# Novel cell-based in vitro screen to identify small-molecule inhibitors against intracellular replication of *Cryptococcus neoformans* in macrophages

**DOI:** 10.1016/j.ijantimicag.2016.04.018

**Published:** 2016-07

**Authors:** Sweta Samantaray, Joao N. Correia, Mariam Garelnabi, Kerstin Voelz, Robin C. May, Rebecca A. Hall

**Affiliations:** Institute of Microbiology and Infection, School of Biosciences, University of Birmingham, Birmingham, UK

**Keywords:** *Cryptococcus neoformans*, Macrophages, Intracellular proliferation, Drug screening, Anticryptococcal therapy, Fendiline

## Abstract

The fungal pathogen *Cryptococcus neoformans* poses a major threat to immunocompromised patients and is a leading killer of human immunodeficiency virus (HIV)-infected patients worldwide. Cryptococci are known to manipulate host macrophages and can either remain latent or proliferate intracellularly within the host phagocyte, a favourable niche that also renders them relatively insensitive to antifungal agents. Here we report an attempt to address this limitation by using a fluorescence-based drug screening method to identify potential inhibitors of intracellular proliferation of *C. neoformans.* The Prestwick Chemical Library^®^ of FDA-approved small molecules was screened for compounds that limit the intracellular replication of a fluorescently-tagged *C. neoformans* reference strain (H99-GFP) in macrophages. Preliminary screening revealed 19 of 1200 compounds that could significantly reduce intracellular growth of the pathogen. Secondary screening and host cell cytotoxicity assays highlighted fendiline hydrochloride as a potential drug candidate for the development of future anticryptococcal therapies. Live cell imaging demonstrated that this Ca^2+^ channel blocker strongly enhanced phagosome maturation in macrophages leading to improved fungal killing and reduced intracellular replication. Whilst the relatively high dose of fendiline hydrochloride required renders it unfit for clinical deployment against cryptococcosis, this study highlights a novel approach for identifying new lead compounds and unravels a pharmacologically promising scaffold towards the development of novel antifungal therapies for this neglected disease.

## Highlights

• Screening of inhibitors against intracellular survival of *Cryptococcus neoformans* is presented.
• Ca^2+^ channel blocker fendiline hydrochloride is identified as a potential candidate.
• Fendiline triggers phagosomal acidification and intracellular fungal killing.
• Mechanistic studies reveal intracellular calcium rise upon drug treatment.
• Fendiline may be a promising drug scaffold for anticryptococcal therapy.

## 1 Introduction

*Cryptococcus neoformans* is an opportunistic fungal pathogen that can cause fatal infections in immunocompromised individuals. The infection process begins with inhalation of infectious agents (spores or desiccated yeasts) resulting in a primary pulmonary infection, which, in response to immunosuppression, can further disseminate to the central nervous system causing meningitis [1]. Human immunodeficiency virus/acquired immune deficiency syndrome (HIV/AIDS) patients are particularly prone to cryptococcal infections, with an estimated overwhelming disease burden of approximately one million cases of cryptococcal meningitis (CM) per year [2], [3]. The highest incidence of CM-related deaths in HIV-positive patients occurs in sub-Saharan Africa, with an associated mortality of 70% [2]. Despite the advent of highly active antiretroviral therapy, CM-related mortality remains prevalent among HIV/AIDS patients in developing regions [4], [5].

Alveolar macrophages form the first line of defence against *Cryptococcus*, however the fungus is able to survive and replicate within the macrophage phagosome following internalisation [6], [7], [8]. The exact mechanisms facilitating intracellular survival of the pathogen within phagosomes are not fully understood, but recent data suggest that the process of phagosomal maturation is subtly perturbed leading to reduced antimicrobial activity in this compartment [9] and phagolysosomal damage [10]. Continued replication of the fungus eventually leads to lysis of the host macrophage or non-lytic release of the pathogen by a process termed vomocytosis [11], [12].

Current World Health Organization (WHO) guidelines for CM management in AIDS patients recommend ‘gold-standard’ combination therapy with amphotericin B (AmB) and 5-flucytosine (5-FC) followed by lifelong maintenance with fluconazole (FLU) [13], [14]. However, the need for intravenous dosing together withsignificant clinical toxicity and thus a requirement for therapeutic monitoring, particularly for optimising 5-FC dosing in patients with renal impairment, has limited deployment of this approach in resource-limited settings [15], [16], [17]. Therefore, novel and effective alternatives to these mainstay anticryptococcal drugs are needed. Compounds that drive clearance of cryptococci from the intracellular niche offer a powerful alternative approach to treating cryptococcosis and may hold promise as adjunct therapy to use alongside existing antifungals.

**Fig. 1:**
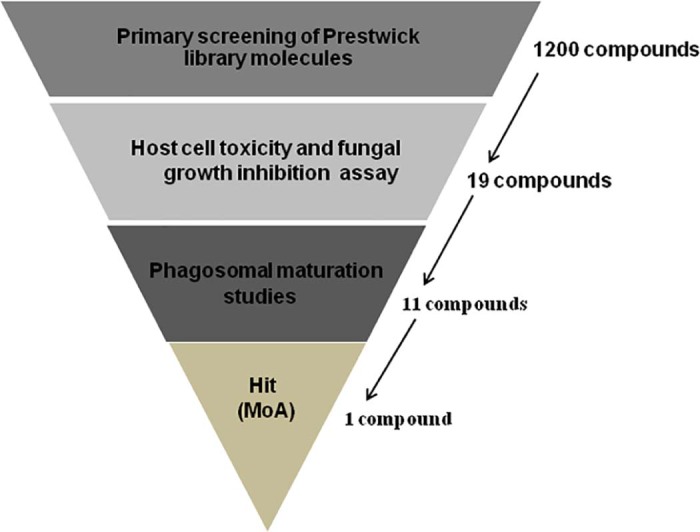
Screening strategy for library molecules. A total of 1200 US Food and Drug administration (FDA)-approved small molecules were screened for intracellular growth inhibition of *Cryptococcus neoformans* in macrophages. The primary screen yielded 19 active compounds that were further probed for host cell cytotoxicity and fungal growth inhibition. Eleven compounds were shortlisted for phagosomal maturation screening, which identified only one promising drug hit candidate, D9 (fendiline hydrochloride). Finally, the mechanism of action of fendiline was investigated. MoA, mode of action.

To identify such compounds, we have employed a high-throughput fluorescence-based screening approach to probe 1200 US Food and Drug Administration (FDA)-approved small molecules for their ability to inhibit intracellular proliferation of *C. neoformans* in murine macrophages (Fig. 1). Shortlisted compounds were further probed for host cell cytotoxicity and antifungal activity, and lead molecules were validated *in vitro* by intracellular killing assays, leading to the identification of fendiline hydrochloride as a potential candidate compound. Finally, we demonstrated that fendiline hydrochloride improves the phagosomal maturation rate and thus facilitates killing of *C. neoformans* within the host cell.

## 2 Materials and methods

### 2.1 Yeast cells and growth conditions

All reagents were purchased from Sigma unless otherwise stated. Green fluorescence protein (GFP)-expressing *C. neoformans* serotype A strain (H99-GFP) and *Cryptococcus gattii* serotype B strain (R265-GFP) were used for this study [18] and were grown overnight in YPD medium (2% glucose, 1% peptone and 1% yeast extract) on a rotator revolving at 240 rpm at 25 °C prior to all experiments.

### 2.2 Macrophage cell line culture

Cells from the murine macrophage-like cell line J774A.1 were used between passages 4 and 14 after thawing and were cultured in Dulbecco's modified Eagle's medium (DMEM) supplemented with 2 mM l-glutamine, 100 U/mL penicillin, 100 U/mL streptomycin and 10% foetal bovine serum (FBS) at 37 °C and 5% CO_2_.

### 2.3 Assay development and primary screening assay

The Prestwick Chemical Library^®^ containing approximately 1200 FDA-approved small molecules was accessed via the Birmingham Drug Discovery Facility (University of Birmingham, Birmingham, UK). Shortlisted compounds were repurchased from Sigma-Aldrich unless otherwise mentioned. The drugs were dissolved in dimethyl sulphoxide (DMSO), which served as a negative control, and were used at a final assay concentration of 10 µM, and AmB (final assay concentration 1.25 µg/mL) was used as a positive control. Primary screening of compounds was performed at the Drug Discovery Unit (University of Birmingham) using a Hamilton STAR liquid handling robot (Hamilton Robotics, Bonaduz, Switzerland) integrated with a microplate reader (BMG LABTECH, Ortenberg, Germany) enabling GFP fluorescence measurement. Macrophages (0.25 × 10^5^ cells/well) were seeded in a glass-bottom 96-well plate (Greiner Bio One Ltd., Stonehouse, UK) 18 h before infection. An overnight culture of strain H99-GFP was harvested by centrifugation at 6500 rpm for 2.5 min, washed three times with phosphate-buffered saline (PBS) and opsonised with 5% pooled human serum (not heat-inactivated) for 1 h at room temperature prior to infection. Macrophages were activated with 150 ng/mL phorbol myristate acetate (PMA) for 1 h in DMEM without FBS and were infected with yeast cells [multiplicity of infection (MOI) 10:1] for 2 h at 37 °C. At this point, total GFP fluorescence was measured (*t*_0_′) to control for well-to-well variation in cryptococcal exposure. Then, the medium was aspirated and cells were washed with PBS to remove extracellular yeasts. The GFP fluorescence corresponding to only intracellular yeast was measured (*t*_0_) and medium containing drugs was added to the cells and was incubated for a further 18 h at 37 °C in 5% CO_2_. After 18 h, the GFP fluorescence was measured (*t*_18_′) to determine the total yeast burden. Extracellular yeast was then removed by washing with PBS and a final GFP fluorescence reading (representing intracellular yeast only) was taken (*t*_18_). The total (intracellular plus extracellular) proliferation rate (PR) was quantified as the relative fluorescence ratio of *t*_18_′/*t*_0_′, whilst the intracellular proliferation rate (IPR) was quantified as the ratio *t*_18_/*t*_0_. The statistical parameters signal-to-basal (S/B) ratio, Z′ factor and percent coefficient of variation (%CV) were calculated as follows [19]:

$$Z^{\prime }=1-\left(3\sigma_{n}+3\sigma_{p}\right)/\left|\mu_{n}-\mu_{p}\right|$$

$$S/B=\mu_{n}/\mu_{p}$$

$$\%\;\text{CV}=\sigma_{n}/\mu_{n}\times 100$$

where µ_n_, σ_n_, µ_p_ and σ_p_ are the means (µ) and standard deviations (σ) of the negative and positive controls, respectively.

### 2.4 Secondary screening (phagosomal maturation studies)

Macrophages (0.5 × 10^5^ cells/well) were seeded into a glass-bottom 96-well plate and were infected with serum-opsonised H99-GFP cells (MOI 10:1) following activation with PMA as described above. Where necessary, yeasts were heat-killed at 55–60 °C for 30 min before serum opsonisation and infection as described previously [9]. At 2 h post-infection, medium was replaced with serum-free DMEM supplemented with 5 µM of drugs (equivalent to 0.1% DMSO) or 1.25 µg/mL AmB containing the acidotropic dye 50 nM LysoTracker^®^ Red DND-99 (Invitrogen, Molecular Probes, Waltham, MA). Cells were then taken for live imaging for 18 h.

### 2.5 Cytotoxicity assay

Macrophage cell cytotoxicity of the compounds was tested using the LDH Cytotoxicity Detection Kit (Takara Bio Inc., Kusatsu, Japan) according to the manufacturer's protocol. Macrophages were seeded into 96-well plates at a density of either 0.25 × 10^5^ cells/well or 0.5 × 10^5^ cells/well and were cultured overnight. Medium was replaced with serum-free DMEM containing either 5 µM or 10 µM of drug and cells were incubated at 37 °C in 5% CO_2_ for a further 18 h. Lactate dehydrogenase (LDH) enzyme released into the cell culture supernatant was measured as a colorimetric change after addition of reaction mixture. The plate was read at 490 nm using a FLUOstar Omega Microplate Reader (BMG Labtech) after 10 min incubation at room temperature. 1% Triton X-100 was used as a positive control. Treatments were done in triplicate and the mean absorbance was calculated. The percent cytotoxicity for samples was expressed as a percentage of the positive control (100% for positive control).

### 2.6 Live imaging

Time-lapse imaging was performed at 37 °C using a Nikon TE2000 microscope (Nikon, Tokyo, Japan) in a humidified chamber with 5% CO_2_ with a 20× or 60× objective (Nikon) as indicated. Images were captured every 5 min for 18 h and were analysed using NIS-Elements Advanced Research software (Nikon). The IPR was determined at 12 h (*t*_12_) due to excessive extracellular cryptococcal growth at 18 h precluding measurement at this time point.

### 2.7 Fungal growth inhibition assay

For the fungal growth inhibition assay, 10^5^ yeast cells were added per well in YPD broth supplemented with 5 µM of drug or the respective control (1.25 µg/mL AmB or 0.1% DMSO) in a 48-well plate (Greiner Bio One). Fungal growth kinetics was measured over a 24-h time period by reading the plate at 600 nm every 30 min using a FLUOstar Omega Microplate Reader. Plates were incubated throughout at 25 °C in the plate reader.

### 2.8 Statistical analysis

All statistical analyses were performed using GraphPad Prism software (GraphPad Software Inc., La Jolla, CA) with all data represented as the mean ± standard deviation (S.D.) from at least three independent experiments unless otherwise stated. Data analyses were done using the Mann–Whitney *U*-test, one-way analysis of variance (ANOVA) or Fisher's exact test (two-tailed) as indicated in the respective figure legends.

## 3 Results

### 3.1 Assay development to monitor intracellular proliferation of *C. neoformans* in macrophages and primary screen design

**Fig. 2:**
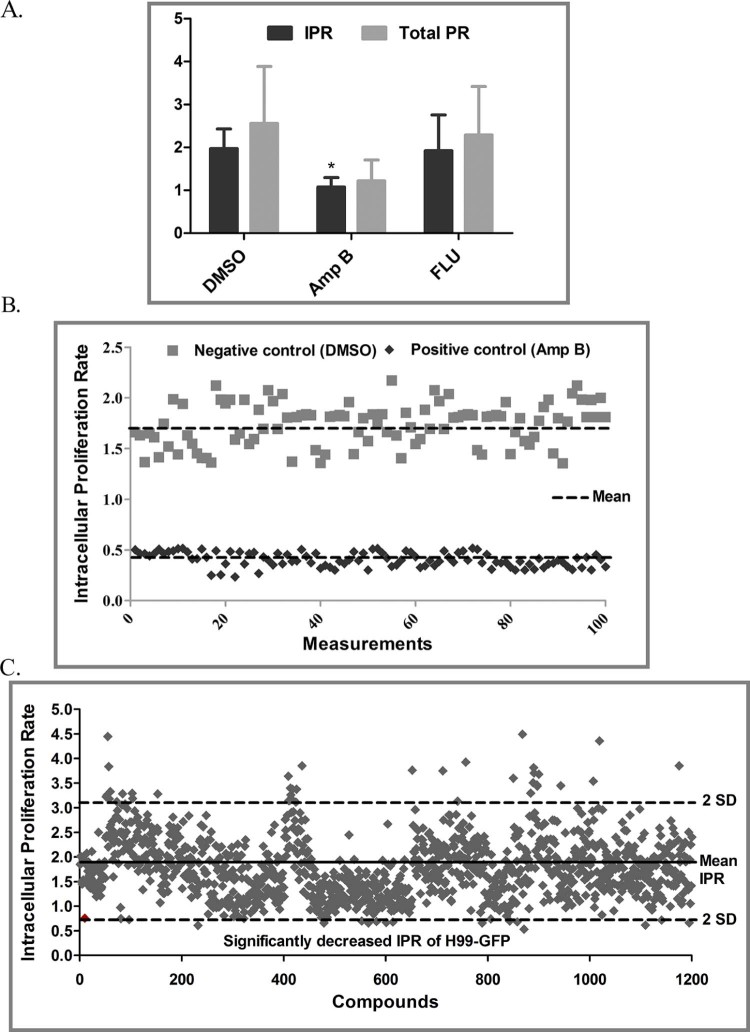
Assay development and determination of Z′ factor for automated screen. (A) Manual intracellular proliferation rate (IPR) evaluation for different drug treatments, including 2% dimethyl sulphoxide (DMSO), 1.25 µg/mL amphotericin B (AmB) and 10 µg/mL fluconazole (FLU). Values represent the mean ± standard deviation (S.D.) from four independent experiments [IPR and proliferation rate (PR) separately analysed by Mann–Whitney *U*-test, *P <* *0.05]. (B) Scatter plot showing the distribution of DMSO-treated (negative control) and AmB-treated (positive control) signal representing the IPR of H99-GFP in macrophages (*n* = 100). (C) Primary screening of 1200 Prestwick Chemical Library^®^ compounds using automatic robot showing the mean IPR of all compounds (solid black line) as well as two S.D. higher and lower IPR values (dashed black lines). IPR value of AmB is indicated in red. (For interpretation of the references to color in this figure legend, the reader is referred to the web version of this article.)

To identify small molecules that inhibit the intracellular growth of *C. neoformans* in macrophages, a high-throughput fluorescence-based automated screen was designed to score replication of a GFP-expressing *C. neoformans* in macrophages (Fig. 1). Macrophages were infected with strain H99-GFP in 96-well plates and the IPR of the yeast was quantified by GFP fluorescence after 18 h following phagocytosis and drug addition. As a proof of concept and in order to establish the accuracy of the assay principle in evaluating intracellular proliferation of H99-GFP under different drug treatments, the IPR and total PR in response to DMSO, AmB and FLU were first assessed manually (Fig. 2A). Whilst the IPR values remained mostly consistent for DMSO (the carrier control) and FLU (which has poor penetration into the phagosome), it decreased significantly for AmB (Fig. 2A), demonstrating that the screening set-up is able to discriminate agents that are active intracellularly from those with only extracellular activity. Therefore, we proceeded with the high-throughput screen using DMSO and AmB as negative and positive controls, respectively, which yielded significantly different IPR values for DMSO and AmB treatments (Fig. 2B). High-throughput screens are typically characterised by a Z′ factor ≥0.5 and high S/B values [19], [20]. According to Zhang et al, an assay with a Z′ value >0 and <0.5 is considered to be marginal but feasible for screening [19]. Both the Z′ factor (0.37) and S/B (4.3) values for our screen fell within the range that is deemed suitable for high-throughput screening [19], [20], and the %CV value of 12% was well below the recommended 20% limit [20] (Table 1; Fig. 2B). Thus, the screening procedure, whilst inherently noisy, appears sufficiently sensitive to identify promising lead compounds.

To exclude the possibility of molecules inhibiting phagocytic uptake of yeasts, the compound library was added after phagocytosis. From the preliminary screening, only those compounds that exhibited a statistically significant decrease in IPR (i.e. 2 S.D. lower than the mean IPR of the total 1200 compounds) were considered as ‘primary hits’ (Fig. 2C). This preliminary screening yielded a total of 19 small molecules that significantly inhibited intracellular growth relative to the DMSO control (data not shown). Compounds that visually exhibited significant macrophage toxicity or with previously reported antimicrobial (including antifungal) activity were further discarded. Compounds that significantly lowered PR values (i.e. showed potent extracellular fungicidal effects) were also excluded (data not shown). After applying these filters, 13 unique molecules that limit *C. neoformans* survival in macrophages were identified. These selected compounds were repurchased from commercial sources for further tests and are denoted as D2–D14 (data not shown).

### 3.2 Validating host cell cytotoxicity and direct antifungal activity of D2–D14 compounds

**Fig. 3:**
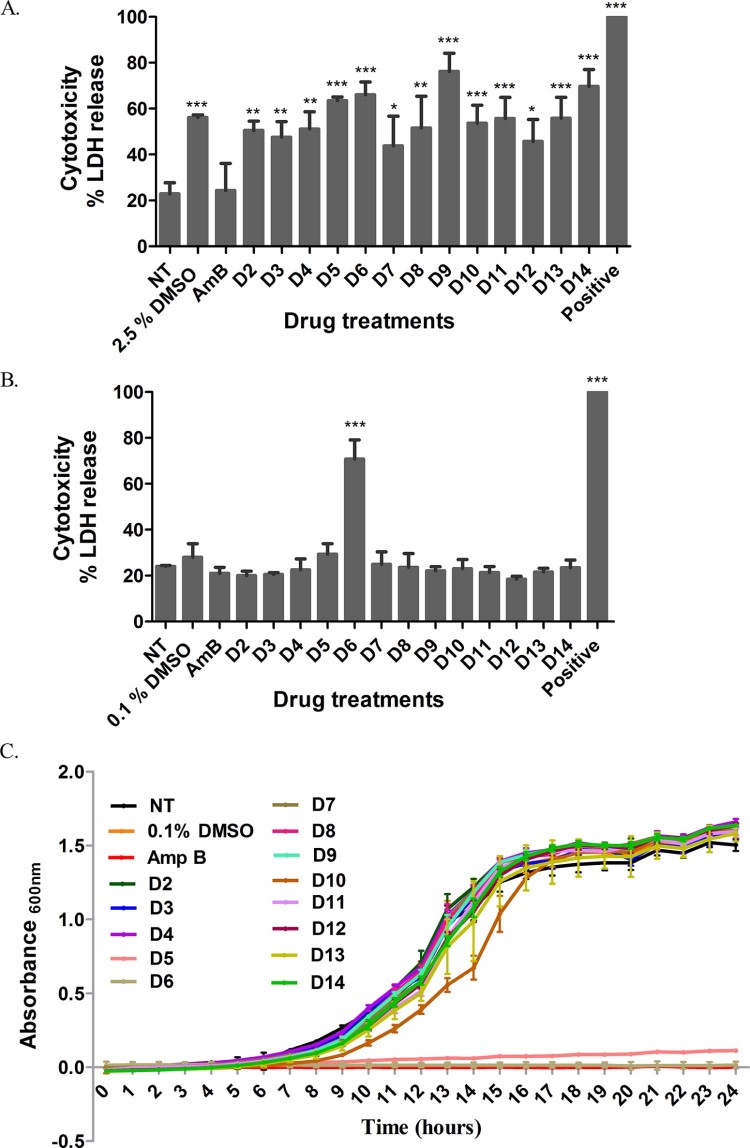
Effect of drug treatments on host cell cytotoxicity and fungal growth inhibition. (A,B) Lactate dehydrogenase (LDH) released from murine macrophages was measured after 18 h of drug treatment with 10 µM drugs (2.5% DMSO) (A) and 5 µM drugs (0.1% DMSO) (B), and 1.25 µg/mL AmB. 0.1% Triton X-100 was used as a positive control. Values represent the mean ± standard deviation (S.D.) from three independent experiments (one-way ANOVA + Dunnett's post-test, *P <* *0.05, **P <* *0.01 and ***P <* *0.001). (C) Effect of 5 µΜ drugs (0.1% DMSO) on growth kinetics of H99-GFP cells. AmB (1.25 µg/mL) was used as a positive control. The growth curve shown is representative of three independent experiments. Values represent mean ± S.D. DMSO, dimethyl sulphoxide; NT, untreated cells.

To confirm the primary screen-specific effects, the 13 selected repurchased compounds were tested under different conditions using a reduced compound concentration (5 µM), lower DMSO content (0.1%) and higher macrophage density. As observed in Fig. 3A, treatment of a macrophage cell density of 0.25 × 10^5^ cells/well with 10 µM compounds (1.25 µg/mL AmB) and a corresponding 2.5% DMSO control yielded a high level of macrophage cytotoxicity. In contrast, treatment with a 5 µM drug dose corresponding to a low DMSO content of 0.1% and higher cell density (0.5 × 10^5^ cells/well) enhanced macrophage survival (Fig. 3B), thereby prompting us to use the latter setting for further investigation.

We validated that the selected doses of the compounds (5 µM and corresponding 0.1% DMSO) were not directly toxic to the fungi. For this, the growth inhibitory activity of the selected compounds was tested against an H99-GFP axenic culture over a period of 24 h (Fig. 3C). Compounds D5 and D6 significantly inhibited fungal growth to the same extent as the positive control AmB and were therefore excluded from further screening. The remainder of the compounds exhibited comparable growth kinetics to the untreated control, suggesting that these compounds do not exhibit direct antifungal activity but rather suppress intracellular growth of the fungus (Fig. 3C).

### 3.3 Secondary screening reveals D9-triggered phagosomal acidification in macrophages

**Fig. 4:**
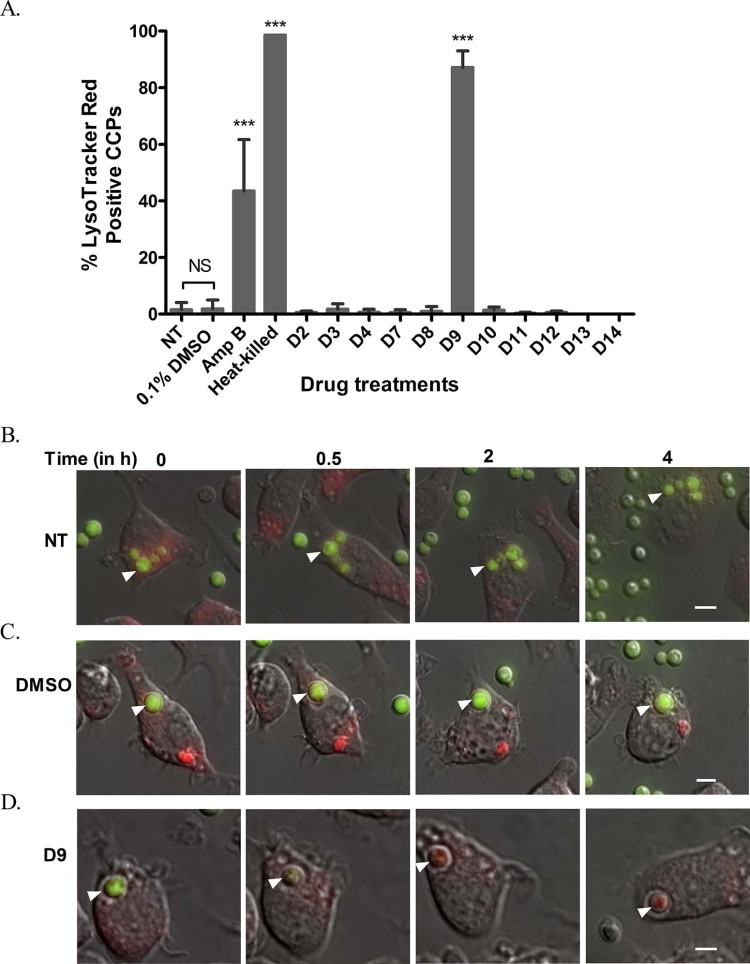
Effect of drugs on macrophage phagosomal maturation. (A) Phagosomal acidification was quantified by staining with LysoTracker^®^ Red and is indicated by percent LysoTracker-positive cryptococcal-containing phagosomes (CCPs) after 18 h treatment with 5 µM drugs. Heat-killed cells served as a positive control. Values represent the mean ± standard deviation (S.D.) collected from observing 300–400 phagosomes at each time point for each treatment across three to five biological repeats (Fisher's exact test, ***P <* *0.001; NS, not significant). (B–D) Merged images of brightfield, green fluorescent protein (GFP) and LysoTracker Red (red fluorescence) taken from time-lapse microscopy experiments at the indicated time points post-phagocytosis on a 60× objective. Images from panels B, C and D represent status of phagosomal acidification by accumulation of LysoTracker Red in CCPs for untreated (NT), DMSO control and D9-treated cells, respectively. Phagocytosed cryptococci are indicated by arrowheads (white). Time-lapse frames are extracted from Movie S1, Movie S3.

To probe how the remaining 11 compounds reduced the intracellular proliferation of *C. neoformans*, their effect on phagosome maturation was assessed. Phagosome maturation is a multistep process eventually resulting in phagolysosome fusion, acidification and enhanced antimicrobial properties [21]. Several intracellular pathogens such as *Mycobacterium tuberculosis*, *Legionella pneumophila*, *Salmonella* Typhimurium and *Leishmania donovani* evade macrophage killing by perturbing the phagosomal maturation process [22], [23]. Recently, we showed that *C. neoformans* is also capable of altering phagosomal acidification in host cells to facilitate intracellular survival [9]. Therefore, we considered the possibility that compounds identified as reducing intracellular growth of cryptococci may do so by improving phagosomal maturation. To test this hypothesis, phagosome acidification was monitored using the fluorescent acidotropic dye LysoTracker Red in response to drug treatment during infection. Whilst live untreated cryptococci-containing phagosomes hardly acquired a LysoTracker-positive signal, almost 100% of the phagosomes in the heat-killed control became LysoTracker-positive over the 18 h period of infection (Fig. 4A). Most of the drug treatments, including the DMSO control, did not yield any significant phagosomal acidification compared with the untreated control (Fig. 4A). Interestingly, however, treatment with drug D9 significantly enhanced phagosomal acidification and yielded a striking 87.14% LysoTracker-positive phagosomes compared with untreated and DMSO controls (Fig. 4A–D; Movie S1, Movie S3). Surprisingly, D9 yielded significantly higher LysoTracker-positive signal than AmB, which is a mainstay anticryptococcal drug with fungicidal activity against cryptococci but generated only 43.53% LysoTracker-positive phagosomes up to 18 h of infection (Fig. 4A; Supplementary Movie S1). Upon careful analysis of time-lapse movies, it was found that the D9-induced acidification events occurred typically within the first 4–5 h of infection (Fig. 4D; Supplementary Movie S3). Conversely, untreated and DMSO-treated phagosomes remained mostly negative at that time point (Fig. 4B,C; Movie S2, Movie S3) and failed to retain a significant amount of LysoTracker-positive signal even 18 h post-infection. Thus, D9 appears to act by enhancing phagosomal maturation and either killing or disabling the intracellular cryptococci.

### 3.4 Drug D9 is active against intracellular cryptococci at 5 µM but loses efficacy at 1 µM

**Fig. 5:**
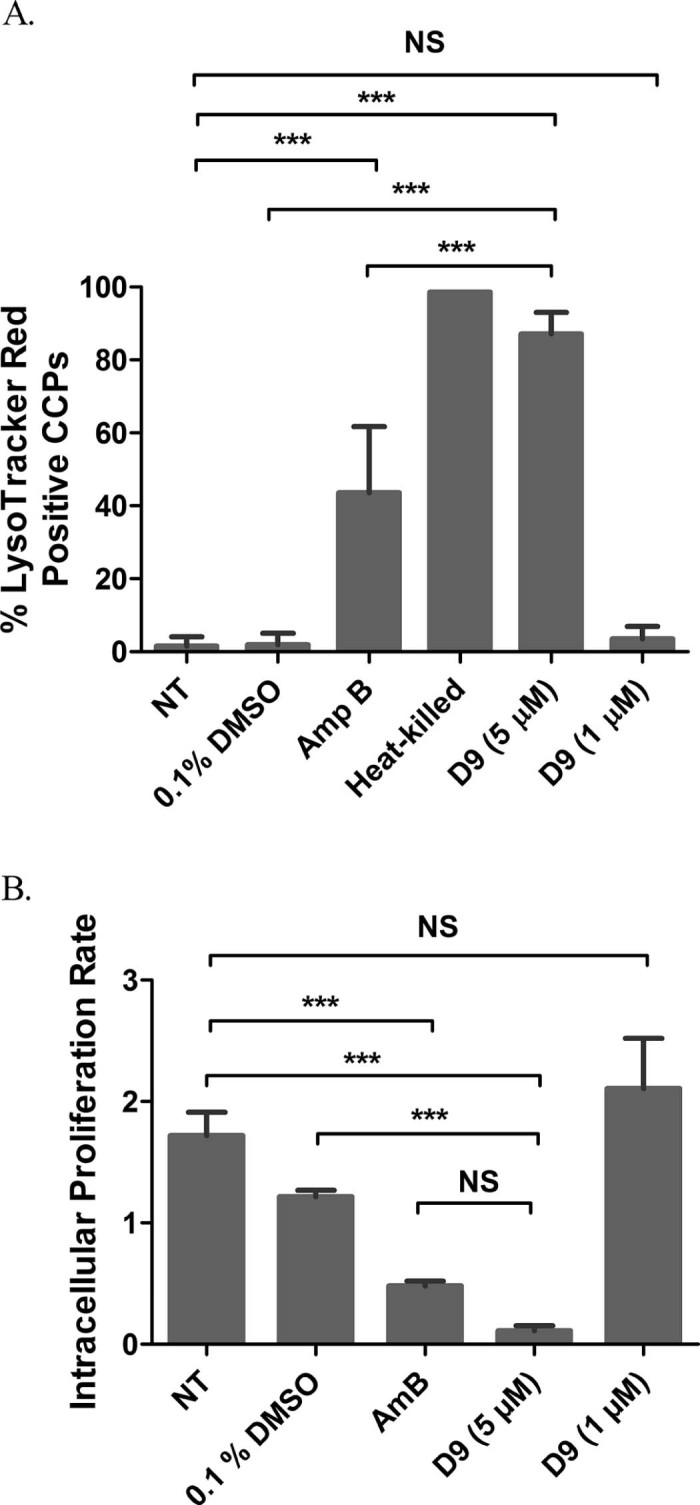
Dose-dependent effect of D9 on phagosomal maturation and intracellular killing. (A) Phagosomal acidification at differential doses of D9 quantified by LysoTracker^®^ Red staining after treatment with the respective drug dose for 18 h. Values represent the mean ± standard deviation (S.D.) collected from observing 300–400 phagosomes at each time point for each treatment across three to five biological repeats (Fisher's exact test, ***P <* *0.001). (B) Effect of respective doses of D9 on intracellular viability of H99-GFP in J774A.1 murine macrophages as quantified from time-lapse movies. The intracellular proliferation rate is calculated as *t*_12_/*t*_0_ ratio, where *t*_12_ and *t*_0_ indicate the number of internalised cryptococci at 12 h and 0 h post-infection, respectively; *n* = 3 (one-way ANOVA + Tukey post-test, ***P <* *0.001; NS, not significant). NT, untreated; DMSO, dimethyl sulphoxide; AmB, amphotericin B; CCP, cryptococcal-containing phagosome.

Next, we were prompted to analyse the efficacy of D9 at a lower dose of 1 µM that would be ideal for clinical applications. However, D9 failed to retain its activity upon lowering the drug dose and the LysoTracker-positive signal significantly dropped to 3.58% compared with that of the 5 µM dose (Fig. 5A). Furthermore, the effect of D9 at both doses on intracellular proliferation of *C. neoformans* in macrophages was assessed from time-lapse movies until 12 h post infection (Fig. 5B). Congruently with its effect on phagosomal acidification, a 5 µM dose of D9 significantly reduced the IPR of H99-GFP cells in macrophages compared with untreated and DMSO controls. Conversely, D9 failed to inhibit intracellular growth of *Cryptococcus* at the lower dose of 1 µM and retained an IPR level comparable with the untreated control, suggesting loss of efficacy at this dose (Fig. 5B). This strong threshold effect itself reflects a requirement for a minimum signalling flux to activate the phagosome maturation pathway in order to successfully kill intracellular cryptococci.

To test whether the activity of D9 is species-specific, the effect of D9 on phagosomal acidification against the other main pathogenic cryptococcal species, *C. gattii* (R265-GFP), was also tested. As with *C. neoformans*, D9 effectively increased LysoTracker-positivity of *C. gattii* phagosomes, an effect that dropped significantly with reduced drug dosage (Supplementary Fig. S1), suggesting that this drug is effective against both human-infective species of *Cryptococcus*.

Unblinding the screening compounds revealed D9 to be fendiline hydrochloride, a known inhibitor of l-type Ca^2+^ channels that reportedly increases the intracellular Ca^2+^ concentration [Ca^2+^]_i_ via calcium release from endoplasmic reticulum stores [24], [25]. Therefore, to assess the intracellular calcium flux in macrophages in response to fendiline, drug concentrations ranging from 1 to 20 µM were tested to check the concentration-dependent effects previously reported for other cell types with this drug [24], [25], [26] (see Supplementary method). Interestingly, fendiline barely had an effect on [Ca^2+^]_i_ at a concentration of 1 µM compared with the DMSO control (Supplementary Fig. S2A), but the [Ca^2+^]_i_ markedly increased at 5 µM or 20 µM (Supplementary Fig. S2A). Thus, this sharp concentration threshold is likely attributable to the differential efficacy observed between 1 µM and 5 µM. Furthermore, to investigate whether the rise in [Ca^2+^]_i_ upon fendiline treatment is mainly due to discharge of Ca^2+^ from intracellular stores, we tested whether the inhibitor thapsigargin specifically blocking endoplasmic reticulum Ca^2+^ pumps could alleviate the rise in [Ca^2+^]_i_ observed with fendiline [25], [26] (see Supplementary method). Indeed, exposing the cells to 0.5 µM thapsigargin caused a significant drop in [Ca^2+^]_i_ compared with the cells exposed to only 5 µM fendiline (Supplementary Fig. S2B). These results suggest that fendiline induces a Ca^2+^ signalling flux in J774A.1 macrophages that involves Ca^2+^ influx to the cytoplasm from intracellular stores.

## 4 Discussion

Considering the poor status of current anticryptococcal drugs, new treatment options for cryptococcosis are much needed. Studies from several groups have attempted to address this and have mostly focused on drugs that act on extracellular, rather than intracellular, cryptococci [27], [28]. Recently, Butts et al screened the Prestwick library of FDA-approved molecules to identify compounds with direct fungicidal activity against *C. neoformans* based on loss of cellular integrity and thereby release of intracellular enzyme adenylate kinase [16]. Although our screening assay utilised the same compound library, it is complementary in approach since it was designed to identify only those molecules that induce intracellular killing, excluding all molecules with direct antifungal activity (including those identified by Butts et al). Importantly, there are no previously reported screens designed to identify adjunct therapies that may augment macrophage-based killing of cryptococci. Therefore, we developed a simple approach to screen the Prestwick compound library by infecting macrophages with the H99-GFP strain and quantifying the GFP fluorescence as an index for intracellular proliferation. Similar approaches to probe intracellular replication using GFP expression have been successfully applied for other intracellular pathogens such as *M. tuberculosis* [29] and *Leishmania* [30]. Intracellular screens are technically challenging, as reflected by the relatively poor Z′ score and S/B ratio of our screen, and thus carry a high false-negative rate. However, our screen was supported by a valid %CV value and the fact that it identified the current frontline anticryptococcal drug (AmB) included in the library. However, we note that there is a possibility that additional anticryptococcal compounds within the collection may have been missed by our screening strategy. Nonetheless, the screen succeeded in identifying a small set of lead compounds, at least one of which appears to be a potential drug candidate.

The screening identified a novel compound D9 (fendiline hydrochloride), an l-type calcium-channel blocker widely used in the treatment of angina [25], [31]. Notably, D9 does not exhibit a direct antifungal effect against *C. neoformans*, but instead triggers significant phagosomal acidification in host macrophages. Fendiline is thought to act by transiently elevating [Ca^2+^]_i_ in certain cell types via release from intracellular endoplasmic reticulum calcium stores [25], [26]. In agreement, we observed an increase in [Ca^2+^]_i_ levels at doses of fendiline above 5 µM, which is inhibited by blocking calcium release from the endoplasmic reticulum with thapsigargin. Interestingly, a previous study by Huang et al showed that a 1 µM dose of fendiline barely triggered any significant increase in [Ca^2+^]_i_ above baseline [25]. This may explain the strong threshold effect at 5 µM, but almost none at the 1 µM drug dose. Considering that intracellular calcium dynamics are altered during cryptococcal infection [9], it is tempting to speculate that fendiline may re-adjust intracellular calcium signalling to allow full phagosome maturation and thus fungal killing, a hypothesis that would be worthy of future investigation in appropriate animal models.

Pharmacologically, fendiline offers some attractive properties relevant for *C. neoformans* infection. Its physiochemical structure comprises a highly lipophilic region linked to a weak base, which enables accumulation in intracellular acidic compartments such as the phagolysosome [32], [33]. In addition, it is known to be an FDA-approved neuroprotective drug and is included in the Neurodegeneration Drug Screening Consortium of 1040 compounds, most of which have access to the blood–brain barrier [34]. This makes the drug scaffold relevant for the treatment of CM infections [35]. However, fendiline itself appears only to be active in vitro at concentrations significantly higher than the typical serum level of 0.6 µM following oral administration [36]. Although this makes fendiline itself unfit for clinical translation, the drug scaffold still offers scope for potential optimisation in the future to improve either efficacy or bioavailability. Alternatively, existing FDA-approved compounds with structural similarity to fendiline may provide promising lines of investigation that would not necessitate extensive pre-clinical testing prior to clinical trials. Importantly, previous studies have highlighted a role for calcium-channel blockers in inhibition of drug efflux pumps and drug resistance in *M. tuberculosis* infection, which shares a similar intraphagosomal lifestyle [37], [38], and thus it is possible that fendiline-derived compounds may have wide applicability.

In conclusion, this work demonstrates an effective screening platform using a whole-cell-based approach to identify inhibitors against intracellular proliferation of *C. neoformans* that can be potentially applied to other intracellular pathogens. We identified a novel off-patent drug, fendiline hydrochloride, that itself is not fungicidal to *C. neoformans* but potently manipulates intraphagosomal replication and viability within the cell. Taken together, these findings propose a role for calcium-channel blockers as potential inhibitors of intracellular survival of *C. neoformans* in infected macrophages and therefore represent a promising strategy for future anticryptococcal drug design and therapy.

## Figures and Tables

**Table 1:** Summary of quality control parameters for screening assay (*n* = 100).

Statistical parameter	Value
Z′ factor	0.37
S/B ratio	4.3
%CV	12.01

## Appendix Supplementary data

Fig. S1 Dose-dependent effect of D9 on phagosomal acidification of macrophages infected with *Cryptococcus gattii* R265-GFP. Phagosomal acidification at differential doses of D9 quantified by LysoTracker^®^ Red staining after treatment with respective drug dose for 18 h. Values represent the mean ± standard deviation (S.D.) collected from observing 300–400 phagosomes (164 phagosomes for heat-killed) at each time point for each treatment across three biological repeats (Fisher's exact test, ****P *<* *0.001). Fig. S1

Fig. S2 Mechanistic studies of D9 (fendiline) in J774A.1 macrophages. (A) Concentration-dependent plot of fendiline compared with 0.1% dimethyl sulphoxide (DMSO) (control) on [Ca^2+^]_i_ level in macrophages. Cells were stained with fluorescent intracellular Ca^2+^ probe Fura-2 AM (5 µΜ) before exposure to DMSO and indicated concentrations of fendiline. [Ca^2+^]_i_ was quantified by fluorescence ratio of excitation at 340 nm and 380 nm and emission at 510 nm. Values represent the mean ± standard error of the mean (SEM) from at least three experiments comprising 30 cells. (B) Effect of intracellular Ca^2+^ stores on fendiline-induced [Ca^2+^]_i_ rise in macrophages. Fendiline (5 µM) and thapsigargin (0.5 µM), an inhibitor for endoplasmic reticulum Ca^2+^ stores, were added in calcium-containing media. Values represent the mean ± SEM from at least three experiments comprising 65 cells (Mann–Whitney *U*-test, ****P* ≤ 0.001). Fig. S2

Movie S1 Lack of phagosomal acidification inside *Cryptococcus*-containing phagosome for untreated macrophage. Time-lapse fluorescent channel merged [brightfield, green fluorescent protein (GFP) and LysoTracker^®^ Red] video showing a cryptococcal spore (GFP) phagocytosed by untreated J774A.1 macrophage without any accumulation of LysoTracker Red dye (in red) inside the macrophage phagosome until 4.5 h post infection. Scale bar, 5 µm. Movie S1

Movie S2 Lack of phagosomal acidification inside *Cryptococcus*-containing phagosome for dimethyl sulphoxide (DMSO)-treated macrophage. Time-lapse fluorescent channel merged [brightfield, green fluorescent protein (GFP) and LysoTracker^®^ Red] video showing a cryptococcal spore (GFP) phagocytosed by J774A.1 macrophage exposed to 0.1% DMSO without any accumulation of LysoTracker Red dye (in red) inside macrophage phagosome until 4.5 h post infection. Scale bar, 5 µm. Movie S2

Movie S3 Lack of phagosomal acidification inside *Cryptococcus*-containing phagosome for dimethyl sulphoxide (DMSO)-treated macrophage. Time-lapse fluorescent channel merged [brightfield, green fluorescent protein (GFP) and LysoTracker^®^ Red] video showing a cryptococcal spore (GFP) phagocytosed by J774A.1 macrophage exposed to 5 µM D9 with intense accumulation of LysoTracker Red dye (in red) inside macrophage phagosome until 4.5 h post infection. Scale bar, 5 µm. Movie S3

Appendix S1 Supplementary method. Appendix S1
