# Supplementary figures and images for: Novel cell-based in vitro screen to identify small-molecule inhibitors against intracellular replication of Cryptococcus neoformans in macrophages
Source: Int J Antimicrob Agents. 2016 Jul;48(1):69–77. doi: 10.1016/j.ijantimicag.2016.04.018 (PMC4942879; doi:10.1016/j.ijantimicag.2016.04.018)

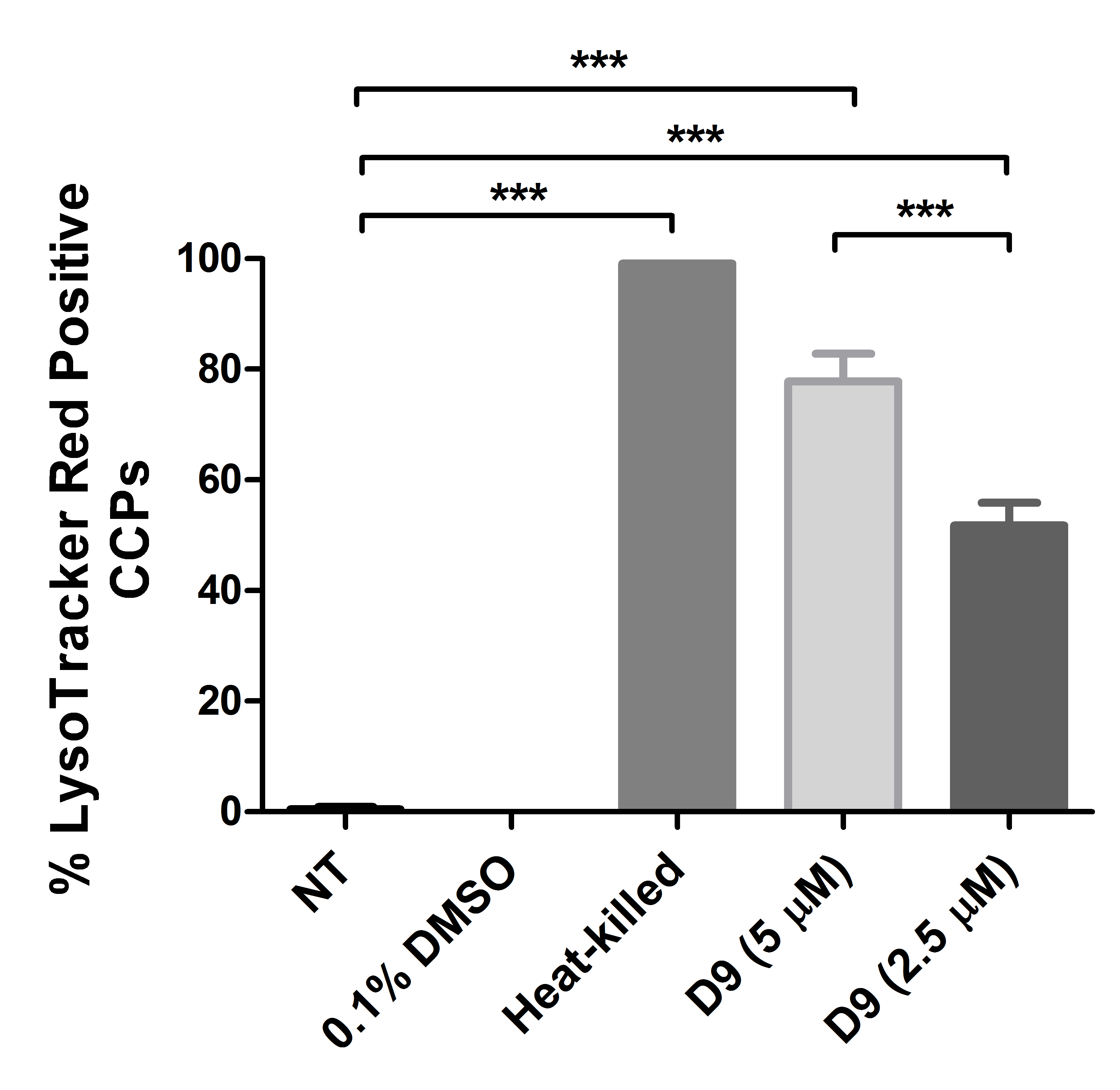

Supplement: Fig. S1 — Dose-dependent effect of D9 on phagosomal acidification of macrophages infected with Cryptococcus gattii R265-GFP. Phagosomal acidification at differential doses of D9 quantified by LysoTracker® Red staining after treatment with respective drug dose for 18 h. Values represent the mean ± standard deviation (S.D.) collected from observing 300–400 phagosomes (164 phagosomes for heat-killed) at each time point for each treatment across three biological repeats (Fisher's exact test, ***P < 0.001). [file mmc1.zip › mmc1.tif]

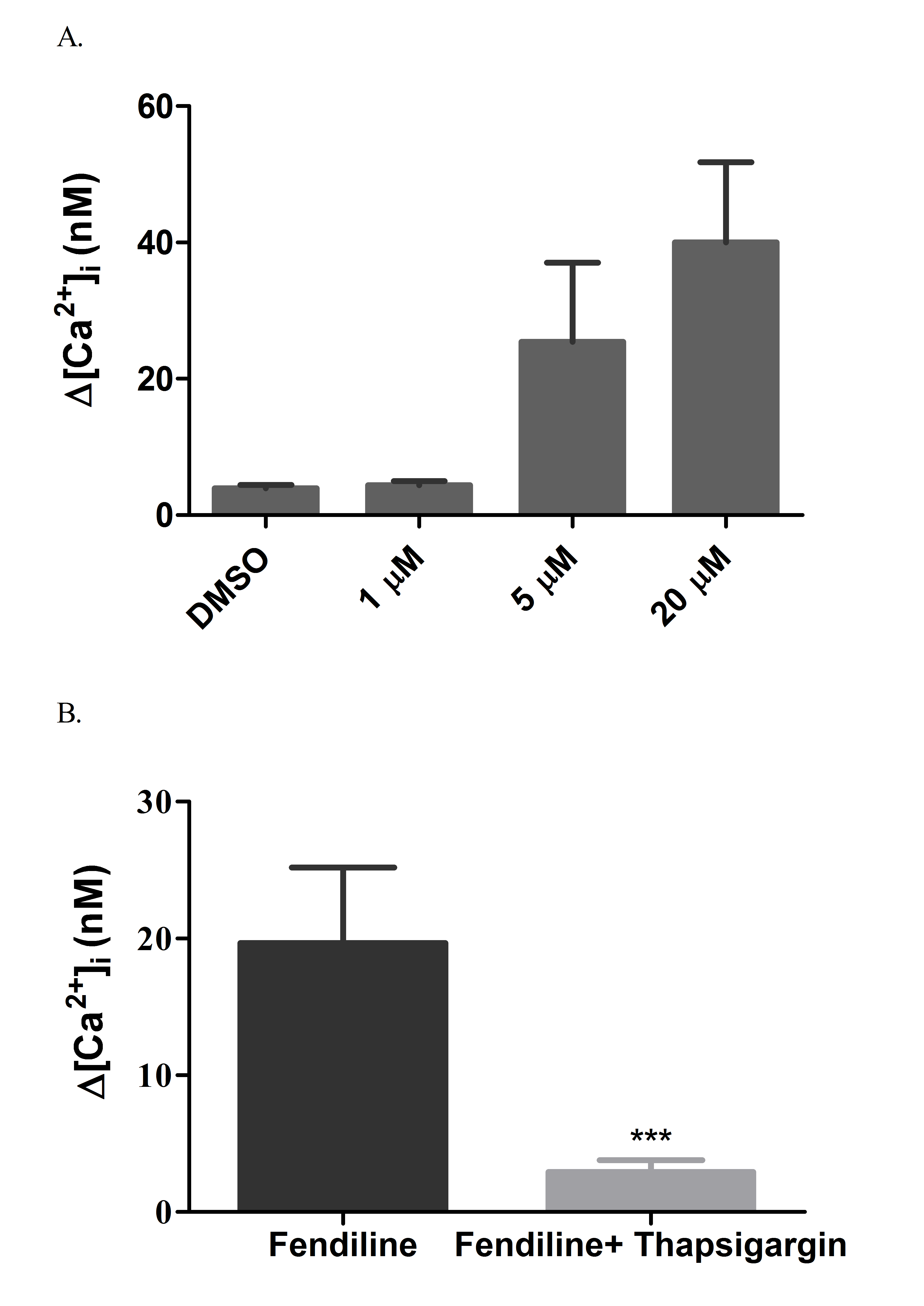

Supplement: Fig. S2 — Mechanistic studies of D9 (fendiline) in J774A.1 macrophages. (A) Concentration-dependent plot of fendiline compared with 0.1% dimethyl sulphoxide (DMSO) (control) on [Ca2+]i level in macrophages. Cells were stained with fluorescent intracellular Ca2+ probe Fura-2 AM (5 µΜ) before exposure to DMSO and indicated concentrations of fendiline. [Ca2+]i was quantified by fluorescence ratio of excitation at 340 nm and 380 nm and emission at 510 nm. Values represent the mean ± standard error of the mean (SEM) from at least three experiments comprising 30 cells. (B) Effect of intracellular Ca2+ stores on fendiline-induced [Ca2+]i rise in macrophages. Fendiline (5 µM) and thapsigargin (0.5 µM), an inhibitor for endoplasmic reticulum Ca2+ stores, were added in calcium-containing media. Values represent the mean ± SEM from at least three experiments comprising 65 cells (Mann–Whitney U-test, ***P ≤ 0.001). [file mmc2.zip › mmc2.tif]
