# Supplementary material for: Novel cell-based in vitro screen to identify small-molecule inhibitors against intracellular replication of Cryptococcus neoformans in macrophages
Source: Int J Antimicrob Agents. 2016 Jul;48(1):69–77. doi: 10.1016/j.ijantimicag.2016.04.018 (PMC4942879; doi:10.1016/j.ijantimicag.2016.04.018)
Supplement: Appendix S1 — Supplementary method. [file mmc6.docx]

**Supplementary method**

This supplementary method section features extended information on the methods provided in the main article.

**Calcium imaging.** The ratiometric fluorescent Ca^2+^ indicator Fura-2 AM (Invitrogen, Molecular Probes) was used to quantify intracellular calcium levels ([Ca^2+^]_i_) in macrophages based on its dissociation constant (K_d_) of 140 nM. Macrophages (0.5 × 105 cells/well) were seeded onto sterile 22 mm × 32 mm coverslips (Thermo Fisher Scientific, #12353128) and were cultured at 37 °C in 5% CO_2_. Coverslips were washed with serum-free Dulbecco’s modified Eagle’s medium (DMEM) before imaging, were mounted on a perfusion chamber and the cells were incubated with 5 μM Fura-2 AM for 45 min at 37 °C. Cells were incubated in dye-free medium for a further 15 min to allow full de-esterification of the dye. Ratiometric measurements were obtained for individual cells under continuous perfusion (flow rate adjusted to 1 mL/min) on an Olympus IX71 microscope using a 40× UPLSAPO objective (Olympus IX71; Olympus, UK). Fluorescence excitation was cycled between 340 ± 5 nm and 380 ± 5 nm through an OptoScan monochromator coupled to an OptoSource xenon arc lamp (Cairn Research, UK) and respective emissions were recorded at 510 ± 20 nm using an Evolve 512 EMCCD camera (Photometrics, USA). MetaFluor^®^ software (Molecular Devices, USA) was used for image acquisition and calculating emission ratios. In situ calibration of intracellular calcium in J774A.1 monolayers was performed as described [[1](#_ENREF_21)] by recording Fura-2 fluorescence as above in calcium-saturated (2.5 mM CaCl_2_ + 10 μM ionomycin) and calcium-free (10 mM EGTA + 10 μM ionomycin) conditions, and subsequent fluorescence ratios were used to obtain [Ca^2+^]_i_ values using the Grynkiewicz equation [[1](#_ENREF_21)]:

[Ca^2+^]_i_ = K_d_ * (R – R_min_) / (R_max_ – R) * F_380max_ /F_380min_

where R is the observed fluorescence intensity ratio at 340/380 nm excitation, R_max_ and R_min_ are the intensity ratios of calcium-saturated (maximum) and calcium-free (minimum) indicator, and F_380max_ /F_380min_ is a scaling factor (fluorescence intensity ratio at 380 nm under maximum and minimum conditions).

For [Ca^2+^]_i_ flux measurements, cells were first perfused with serum-free DMEM to record baseline calcium levels and were subsequently exposed to either dimethyl sulphoxide (DMSO) (0.1%) alone or to drug D9. To assess the contribution of intracellular calcium stores to the calcium transients triggered by D9, macrophages were exposed to the drug in the presence or absence of 0.5 μM thapsigargin.

**Reference**

1. Petr MJ, Wurster RD. Determination of in situ dissociation constant for Fura-2 and quantitation of background fluorescence in astrocyte cell line U373-MG. Cell Calcium 1997;21:233–40.
